# Supplementary material for: Feature-Based Molecular Network-Assisted Cannabinoid and Flavonoid Profiling of Cannabis sativa Leaves and Their Antioxidant Properties
Source: Antioxidants (Basel). 2024 Jun 20;13(6):749. doi: 10.3390/antiox13060749 (PMC11200612; doi:10.3390/antiox13060749)
Supplement: Supplementary file 1 [file antioxidants-13-00749-s001.zip › antioxidants-3029752-supplementary.pdf]

antioxidants

Supplementary Material

Feature-based molecular networks-assisted cannabinoids and flavonoids profiling of *Cannabis sativa* leaves and their antioxidant properties

Ling Chen [1], Hong-Ling Li [1,2], Hong-Juan Zhou [1,2], Guan-Zhong Zhang [1,2], Ying Zhang [3], You-Mei Wang [4], Meng-Yuan Wang [1], Hua Yang [1,2,*] and Wen Gao [1,2,*]

[1] State Key Laboratory of Natural Medicines, School of Traditional Chinese Pharmacy, China Pharmaceutical University, Nanjing 211198, China; cl9426464@163.com (L.C.); honglingli111@163.com (H.L.L.); 17775481058@163.com (H.J.Z.); ahyzgz@163.com (G.Z.Z.); wangmengyuan0202@163.com (M.Y.W.)
[2] China National Narcotics Control Commission-China Pharmaceutical University Joint Laboratory on Key Technologies of Narcotics Control, Nanjing 210009, China
[3] Institute of Forensic Science, Ministry of Public Security, No. 17 South Muxidi Lane, Xicheng District, 100038 Beijing, China; ying_zh@126.com (Y.Z.)
[4] Key Laboratory of Drug Monitoring and Control, Drug Intelligence and Forensic Center, Ministry of Public Security, 100193 Beijing, China; youmei_626@163.com (Y.M.W.)
* Correspondence: gw_cpu@126.com (W.G.); Tel.: +86-25-8618-5219 (W.G.); yanghuacpu@126.com (H.Y.); Tel.: +86-25-8618-5190 (H.Y.)

Citation: To be added by editorial staff during production.

Academic Editor: Firstname Lastname

Received: date
Revised: date
Accepted: date
Published: 20 June 2024

Copyright: © 2024 by the authors. Licensee MDPI, Basel, Switzerland. This article is an open access article distributed under the terms and conditions of the Creative Commons Attribution (CC BY) license (https://creativecommons.org/licenses/by/4.0/).

Abstract: *Cannabis sativa* (*C. sativa*) leaves are rich in cannabinoids and flavonoids, which play important antioxidant roles. Since the regional distribution can influence the accumulation of antioxidants in natural products, which in turn affects their activity, this study aimed to investigate the correlation between the chemical composition of *C. sativa* leaves and their geographical origin and antioxidant activity. Firstly, a high-resolution mass spectrometry method assisted by semi-quantitative FBMN (SQFBMN) was established for the characterization and quantitative analysis of *C. sativa* leaves from various regions. Subsequently, antioxidant activity analysis was conducted on 73 batches of *C. sativa* leaves, and partial least squares regression (PLS) model was employed to assess the correlation between the content of cannabinoids and flavonoids in the leaves and their antioxidant activity. A total of 16 cannabinoids and 57 flavonoids were annotated from *C. sativa*, and showed significant geographical distribution with regularity. The content of flavonoid-C glycosides in Sichuan leaves is relatively high, and their antioxidant activity is also correspondingly high. While, the leaves in Shaanxi and Xinjiang were mainly composed of flavonoid-O glycosides, with slightly lower antioxidant activity. A significant positive correlation ($p<0.001$) was found between the total flavonoids and cannabinoids and the antioxidant activity of the leaves, and two flavonoids and one cannabinoid were identified as significant contributors.

Keywords: Flavonoids; Cannabinoids; Quantification; Antioxidation

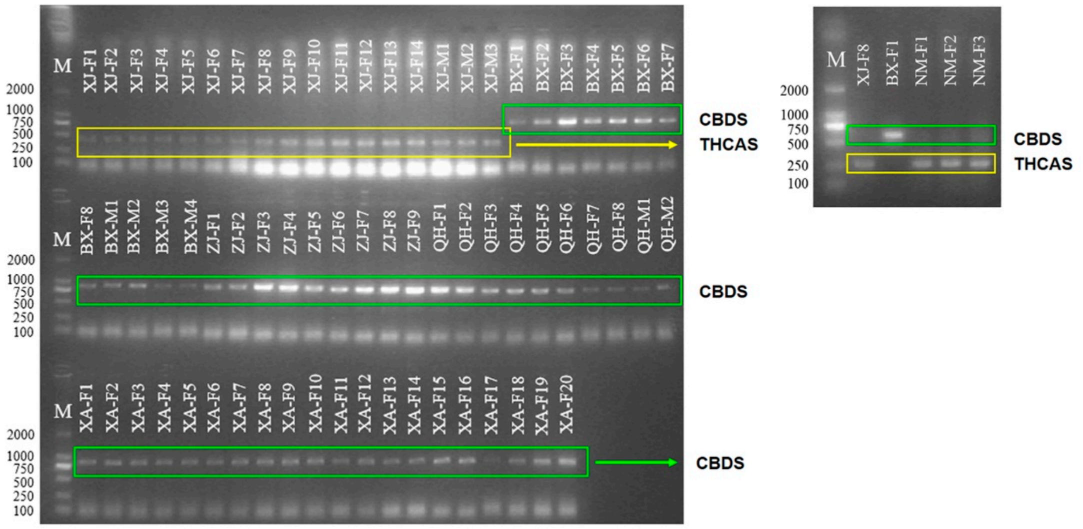

Figure S1. PCR molecular identification results of different batches of *C. sativa* leaves. (XJ: kashgar, Xinjiang Province; BX: Baoxing, Sichuan Province; ZJ: Zhaojue, Sichuan Province; QH: Xi'ning, Qinghai Province; XA: Xi'an, Shaanxi Province; IM: Hulunbuir Inner Mongolia.)

Method: DNA from *C. sativa* leaves was extracted and amplified by PCR. Primer sequences targeting the drug-type THCAS were designed based on a previous report by Tadashi Yamamuro et al [23]. Primer sequences used for THCAS were H-THCAS Fw: 5'-CTGGTTGCTGTCCCATCAAAGTC-3' and H-THCASRv: 5'-GACCATTGCAGTTTCTGGAATTGG-3'. We designed the following primers targeting the fiber-type CBDAS: H-CBDSFw: 5'-GCGGTGGTCATGATTCTGAAGAC-3' and H-CBDSRv: 5'-CGCATACATCCCAACTCCTTCATC-3'. The optimized conditions of the PCR mixture and thermal cycles are shown in Table. Sterilized water was used as a negative control.

Optimized conditions for allele-specific PCR.

	Active *THCAS* single-plex PCR	Active *CBDAS* single-plex PCR	Duplex PCR
PCR mixture (25 μL)			
2XM5 Hi PerplusTaq Hi Fi PCR mix	12.5 μL	12.5 μL	12.5 μL
DNA solution	1 μL	1 μL	1 μL
H-THCASFw	1 μL (10 μM)	-	1 μL (10 μM)
H-THCASRv	1 μL (10 μM)	-	1 μL (10 μM)
H-CBDSFw	-	1 μL (10 μM)	1 μL (10 μM)
H-CBDSRv	-	1 μL (10 μM)	1 μL (10 μM)
Sterilized water	Up to 25 μL	Up to 25 μL	Up to 25 μL
3 step PCR conditions (Time required)	94°C pre denaturation for 4 min; Denaturation at 94°C for 30 s, annealing at 58°C for 45 s, extension at 72°C for 1 min, 35 cycles; Extend at 72°C for 7 min; Store at 4°C		

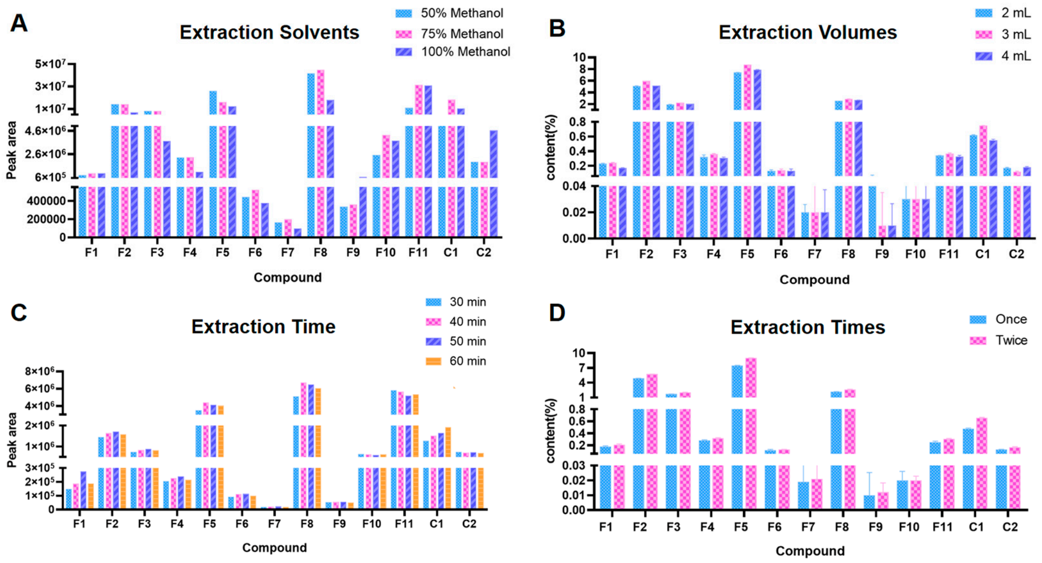

Figure S2. The results of extraction conditions investigated.

(A. Investigation of extraction solvent; B. Investigation of extraction volumes; C. Investigation of extraction time; D. Investigation of extraction times)

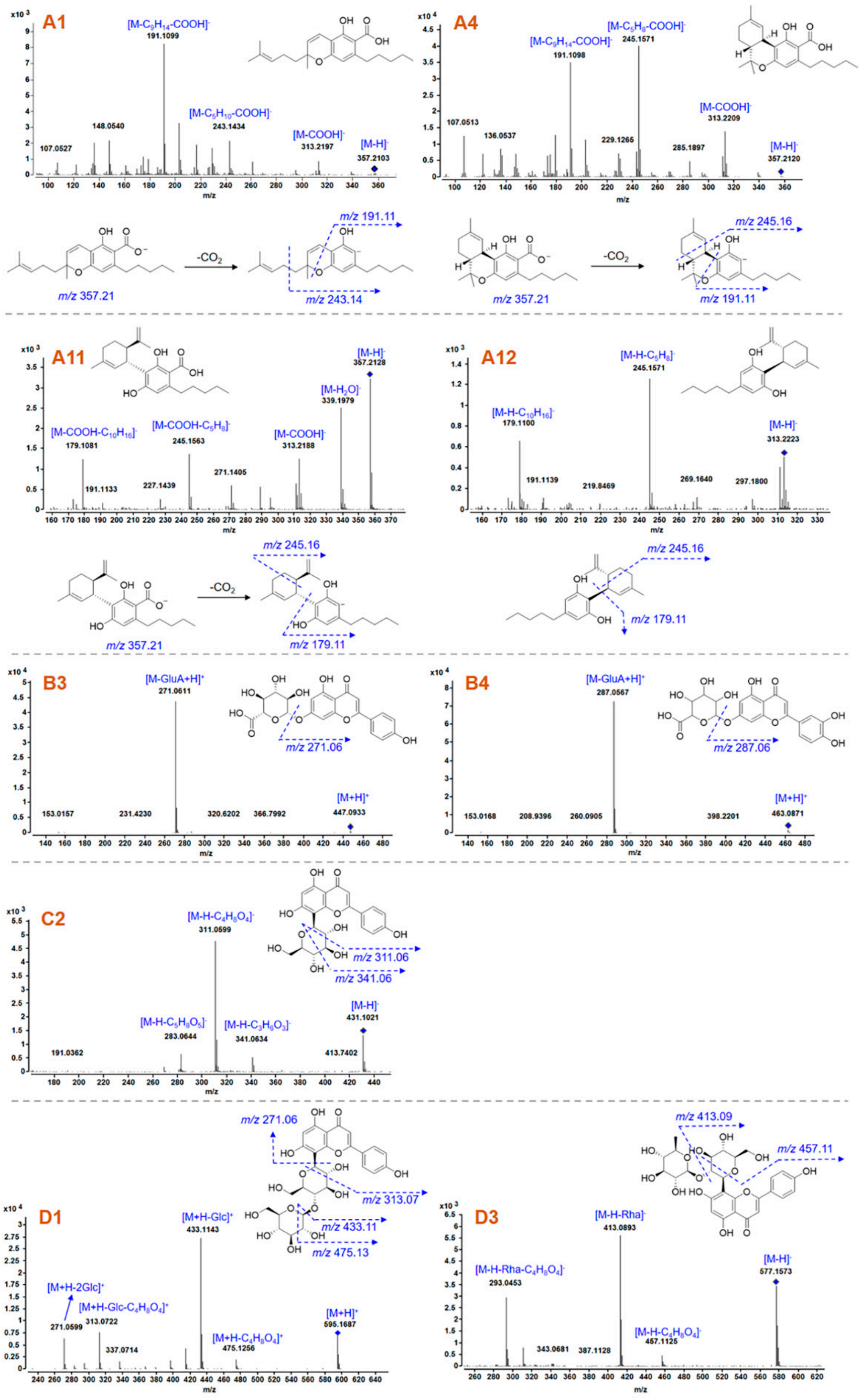

Figure S3. MS/MS spectrum and fragment annotation of reference standards.

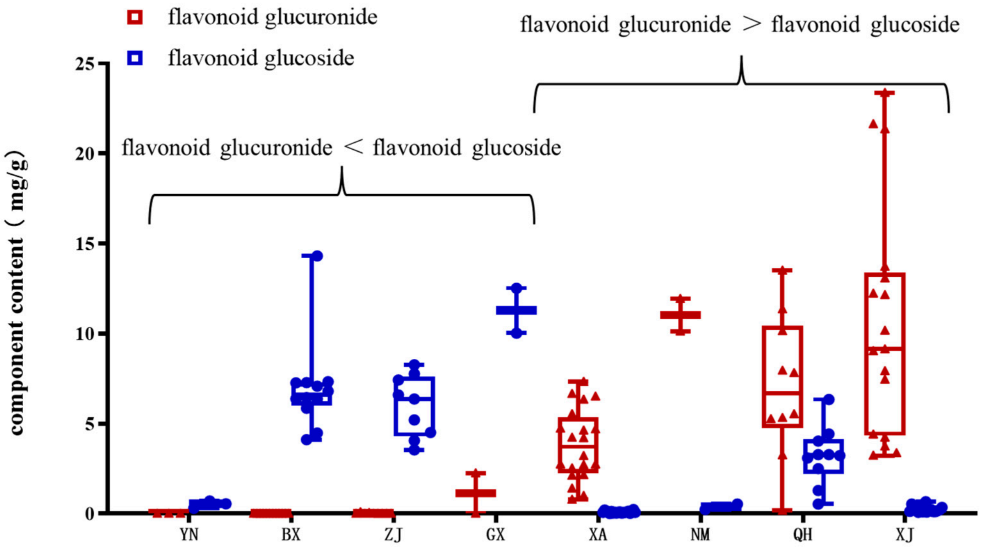

Figure S4-1 Content distribution of total flavonoid glycosides in 8 regions

(YN: Yunnan sample; BX: Sichuan Baoxing sample; ZJ: Sichuan Zhaojue sample; GX: Guangxi Nanning sample; XA: Shaanxi Xi'an sample; NM: Inner Mongolia Hohhot sample; QH: Qinghai Xining sample; XJ: Xinjiang Kashgar sample)

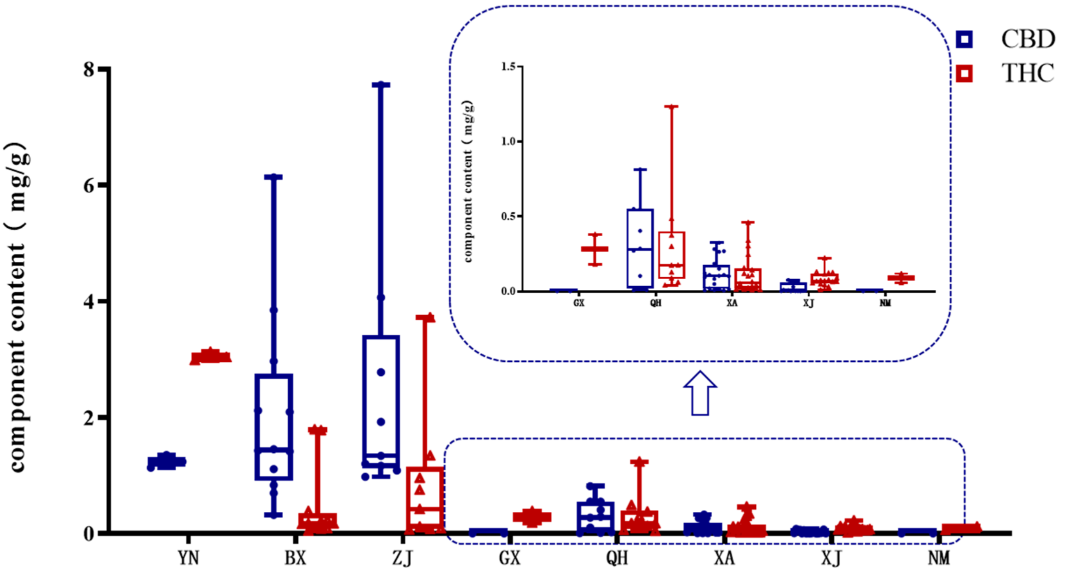

Figure S4-2 Boxplots of cannabinoids in *C. sativa* samples from 8 different regions

(YN: Yunnan sample; BX: Sichuan Baoxing sample; ZJ: Sichuan Zhaojue sample; GX: Guangxi Nanning sample; XA: Shaanxi Xi'an sample; NM: Inner Mongolia Hohhot sample; QH: Qinghai Xining sample; XJ: Xinjiang Kashgar sample)

Table S1. Sample information of *C. sativa* leaves

No.	Source	Amount	Collected time	Type
L01	Yunnan Province (YN)	1 (H)*	2020.07.23	Cultivated
L02-L03	Nanning, Guangxi Autonomous Region (GX)	2 (H)	2019.09.18	Wild
L04-L15	Baoxing, Sichuan Province (SC-B)	12 (8F/4M)	2019.07.02	Cultivated
L16-L24	Zhaojue, Sichuan province (SC-Z)	10 (8F/2M)	2019.07.25	Cultivated
L25-L34	Xining, Qinghai Province (QH)	9 (F)	2019.07.23	Wild
L35-L54	Xi'an, Shaanxi Province (SX)	20 (F)	2019.10.09	Cultivated
L55-L71	Kashgar, Xinjiang Province (XJ)	17 (14F/3M)	2019.10.19	Wild
L72-L73	Huhehaote, Inner Mongolia Autonomous Region (IM)	2 (H)	2019.08.28	Cultivated

* F: Female; M: Male; H: Mix sample

Table S2. ***C. sativa*** **sources for SQFBMN analysis and preparation of mixed samples**

No.	Source	Mixed Sample
BX-F1	Baoxing, Sichuan Province	Cannabis-BX
BX-F2	Baoxing, Sichuan Province	
BX-M3	Baoxing, Sichuan Province	
ZJ-F3	Zhaojue, Sichuan Province	Cannabis-ZJ
ZJ-F4	Zhaojue, Sichuan Province	
ZJ-F7	Zhaojue, Sichuan Province	
NM-F1	Hulunbuir, Inner Mongolia Province	Cannabis-NM
NM-F2	Hulunbuir, Inner Mongolia Province	
NM-F3	Hulunbuir, Inner Mongolia Province	
XA F1-2	Xi'an, Shaanxi Province	Cannabis-XA
XA F7-1	Xi'an, Shaanxi Province	
XJ-F8	Kashgar, Xinjiang Province	Cannabis-XJ
XJ-F9	Kashgar, Xinjiang Province	

Table S3. Results of Different Solvent Extraction Efficiency for *C. sativa* Leaves (Sample L28, Qinghai Province) (n=3, %)

ID	50% Methanol		75% Methanol		100% Methanol	
	Peak area (Mean)	RSD （%）	Peak area (Mean)	RSD （%）	Peak area (Mean)	RSD （%）
F1	7.88×10^{5}	0.44	9.69×10^{5}	1.35	9.40×10^{5}	0.48
F2	1.43×10^{7}	2.05	1.43×10^{7}	6.57	6.92×10^{6}	2.74
F3	8.15×10^{6}	2.76	8.19×10^{6}	5.75	3.73×10^{6}	2.84
F4	2.32×10^{6}	3.06	2.33×10^{6}	4.71	1.06×10^{6}	3.55
F5	2.60×10^{7}	3.70	2.62×10^{7}	4.84	1.23×10^{7}	3.39
F6	4.42×10^{5}	4.39	5.22×10^{5}	6.04	3.77×10^{5}	3.98
F7	1.66×10^{5}	1.64	1.98×10^{5}	2.09	9.76×10^{4}	2.75
F8	4.18×10^{7}	1.88	4.51×10^{7}	0.73	1.79×10^{7}	0.72
IS	3.32×10^{4}	4.81	3.58×10^{4}	1.67	3.78×10^{4}	3.75
F9	3.36×10^{5}	1.04	3.61×10^{5}	1.67	6.29×10^{5}	1.08
F10	2.51×10^{6}	0.57	4.24×10^{6}	0.67	3.75×10^{6}	1.62
F11	1.09×10^{7}	0.39	3.15×10^{7}	0.14	3.09×10^{7}	0.26
C1	5.06×10^{6}	0.61	1.83×10^{7}	0.79	1.04×10^{7}	0.76
C2	1.94×10^{6}	0.31	1.94×10^{6}	3.14	4.63×10^{6}	0.81
SUM	1.15×10^{8}	1.97	1.54×10^{8}	2.87	9.37×10^{7}	2.05

Table S4. Results of Different Solvent Volumes Extraction Efficiency for *C. sativa* Leaves (Sample L28, Qinghai Province) (n=3, %)

ID	2 mL		3 mL		4 mL	
	Amount (Mean, %)	RSD（%）	Contents (Mean, %)	RSD（%）	Contents (Mean, %)	RSD（%）
F1	0.23	0.54	0.24	0.79	0.17	0.43
F2	5.12	2.03	5.96	0.67	5.19	0.21
F3	1.95	2.00	2.24	0.36	2.04	0.80
F4	0.32	2.99	0.36	0.69	0.31	1.09
F5	7.42	2.18	8.78	0.25	7.90	0.81
F6	0.13	1.69	0.14	1.64	0.13	2.74
F7	0.02	0.60	0.02	2.34	0.02	1.72
F8	2.58	0.45	2.90	1.33	2.69	1.07
F9	0.04	3.40	0.01	2.51	0.01	1.66
F10	0.03	1.34	0.03	1.94	0.03	1.86
F11	0.34	0.28	0.37	0.65	0.33	1.40
C1	0.62	0.41	0.75	0.18	0.55	1.32
C2	0.17	0.89	0.12	0.73	0.18	0.95

Table S5. Results of Different Extraction Times Extraction Efficiency for *C. sativa* Leaves (Sample L28, Qinghai Province) (n=3, %)

ID	30 min		40 min		50 min		60 min	
	Peak area (Mean)	RSD（%）	Peak area (Mean)	RSD（%）	Peak area (Mean)	RSD（%）	Peak area (Mean)	RSD（%）
F1	1.49×10^5	1.45	1.87×10^5	0.15	2.75×10^5	0.35	1.88×10^5	2.63
F2	1.46×10^6	2.81	1.64×10^6	0.44	1.71×10^6	1.71	1.58×10^6	2.23
F3	7.36×10^5	1.75	8.38×10^5	1.07	8.83×10^5	0.99	8.18×10^5	2.65
F4	2.06×10^5	2.58	2.28×10^5	1.08	2.39×10^5	1.26	2.15×10^5	1.79
F5	3.50×10^6	1.39	4.40×10^6	1.76	4.13×10^6	0.82	4.04×10^6	1.99
F6	9.30×10^4	1.83	1.12×10^5	2.38	1.13×10^5	2.24	1.00×10^5	0.64
F7	1.80×10^4	2.09	2.05×10^4	1.21	2.28×10^4	2.06	1.95×10^4	1.21
F8	5.12×10^6	1.23	6.74×10^6	0.85	6.49×10^6	1.15	6.05×10^6	3.97
F9	5.24×10^4	0.62	5.47×10^4	0.10	5.62×10^4	1.13	5.20×10^4	5.49
F10	6.36×10^5	1.34	6.33×10^5	0.98	5.99×10^5	0.77	6.30×10^5	1.95
F11	5.82×10^6	2.77	5.66×10^6	0.99	5.18×10^6	0.70	5.35×10^6	0.38
C1	1.27×10^6	1.39	1.52×10^6	0.58	1.65×10^6	0.57	1.92×10^6	0.74
C2	7.40×10^5	1.75	7.05×10^5	0.31	7.26×10^5	0.71	6.96×10^5	0.89

Table S6. Results of Complete Extraction Rate of *C. sativa* Leaves (Sample L28, Qinghai Province) (n=3, %)

ID	Once		Twice		Extract rate of extraction once (%)
	Contents (Mean, %)	RSD（%）	Contents (Mean, %)	RSD（%）	
F1	0.18	1.24	0.21	1.35	85.71
F2	4.89	2.54	5.74	1.92	85.19
F3	1.71	2.89	2.04	2.6	83.82
F4	0.28	1.14	0.32	0.85	87.50
F5	7.51	1.87	9.02	1.91	83.26
F6	0.12	1.24	0.13	0.53	92.31
F7	0.019	1.58	0.021	1.43	90.48
F8	2.17	2.68	2.66	0.96	81.58
F9	0.01	1.53	0.012	0.63	83.33
F10	0.02	0.61	0.02	0.28	100.00
F11	0.25	1.91	0.31	0.57	80.65
C1	0.48	0.77	0.66	0.69	72.73
C2	0.13	0.59	0.17	0.94	76.47
Total	17.769	--	21.313	--	84.85

Table S7. Mass spectrometry parameters of 13 analytes and the IS.

ID	RT (min)	Name	ESI Ion Polarity	Precursor Ion (*m*/*z*)	Product Ion (*m*/*z*)	Q1 (V)	CE (V)	Q3 (V)
F1	3.297	(-)-Epicatechin	Positive	291.2	139.1	-10	-15	-27
F2	4.411	Vitexin-4''-*O*-glucoside	Negative	593.3	293.15	22	36	21
F3	4.697	Vitexin-2''-*O*-rhamnoside	Negative	577.3	293.1	22	36	20
F4	4.869	Vitexin	Negative	431.2	311.2	13	23	21
F5	5.455	Luteolin-7-*O*-glucoside	Negative	447.25	285.2	13	27	19
F6	5.464	Luteolin 7-*O*-glucuronide	Negative	461.2	285.2	14	24	13
F7	7.867	Apigenin-7-*O*-glucoside	Negative	431.1	268.2	13	34	18
F8	8.169	Apigenin-7-*O*-glucuronide	Negative	447.2	271.15	-16	-23	-20
IS	8.505	Xanthotoxol	Positive	203.15	147.2	-11	-23	-29
F9	9.896	Diosmetin 7-*O*-glucoside	Positive	463.2	301.1	-16	-22	-22
F10	13.889	Cannflavin B	Positive	369.2	313.15	-13	-21	-23
F11	15.134	Cannflavin A	Positive	437.3	313.15	-16	-22	-23
C1	15.457	CBD	Positive	315.3	193.25	-11	-25	-20
C2	17.324	Δ^9-THC	Positive	315.3	193.2	-11	-20	-21

Table S8. Investigation of precision, repeatability, stability, and recovery of 13 components from *C. sativa* leave

ID	Intra-day precision (RSD, n=6，%)			Inter-day precision (RSD, n=6, %)			Repeatability	Stability	Accuracy (n=6)	
	Low	Middle	Hight	Low	Middle	Hight	(RSD, n=6, %)	RSD (%)	Recovery (%)	RSD (%)
F1	1.71	0.62	0.82	1.45	2.08	0.94	10.40	3.28	91.00	3.92
F2	1.61	1.43	0.86	4.19	1.47	2.62	8.47	8.69	101.80	7.06
F3	2.69	0.96	1.00	2.75	1.76	2.87	7.19	8.23	104.90	11.38
F4	2.64	1.93	0.44	3.05	1.54	2.29	7.92	6.87	107.00	10.78
F5	1.82	1.76	0.74	2.59	0.63	2.30	10.26	10.06	96.00	11.77
F6	2.93	0.74	0.70	2.49	1.12	2.07	11.04	6.99	103.00	11.80
F7	3.09	1.66	0.75	3.50	3.17	3.52	8.21	5.93	112.00	6.08
F8	0.85	1.23	0.46	1.93	2.77	1.75	8.15	4.16	92.00	7.16
F9	6.85	2.64	0.85	7.14	3.41	1.63	9.21	1.56	99.20	5.82
F10	3.53	0.78	0.92	4.96	1.81	1.37	6.65	1.20	80.00	4.37
F11	5.10	0.81	0.39	4.68	1.68	1.31	6.96	1.42	90.00	7.81
C1	0.94	0.71	0.9	4.84	4.54	3.15	7.81	3.33	101.60	6.30
C2	1.25	0.41	0.51	3.04	4.19	3.00	10.74	2.98	90.00	5.01

Table S9. FBMN-based identification of cannabinoids and flavonoids in *C. sativa* leaves

No.	Formula	RT	*m/z*	Adducts	Diff (ppm)	MS/MS fragments	Identification	Compounds type
A1*	$C_{22}H_{30}O_4$	79.886	359.2206	$[M+H]^+$	-3.03	123.1180;219.1023;341.2106	CBCA	Cannabinoids
			357.2079	$[M-H]^-$	-2.14	179.1074;245.1530;313.2185;339.1964		
A2	$C_{23}H_{34}O_4$	80.746	373.2400	$[M-H]^-$	4.19	191.1089;245.1561;329.2501	CBGAM	Cannabinoids
A3	$C_{20}H_{26}O_4$	73.008	331.1875	$[M+H]^+$	-8.74	191.0690;313.1792	Δ⁹-THCVA	Cannabinoids
			329.1766	$[M-H]^-$	2.32	151.0788;163.0759;175.0858;217.1245;285.1870;311.1688		
A4*	$C_{22}H_{30}O_4$	78.658	359.2206	$[M+H]^+$	-3.03	219.1000;341.2110	Δ⁹-THCA A	Cannabinoids
			357.2079	$[M-H]^-$	-2.14	179.1074;245.1530;313.2185;339.1964		
A5	$C_{21}H_{28}O_4$	73.377	343.2128	$[M-H]^-$	-3.75	150.0737;165.0953;231.1417	Δ⁹-THCA-C4	Cannabinoids
A6	$C_{20}H_{26}O_4$	74.728	329.1776	$[M-H]^-$	-5.35	163.0779;177.0924;202.0972;285.1881;311.1654	CBCVA	Cannabinoids
A7	$C_{21}H_{30}O_4$	76.324	345.2071	$[M-H]^-$	0.1	163.0791;217.1259;301.2169	CBGA-C4	Cannabinoids
A8	$C_{23}H_{32}O_4$	78.044	373.2367	$[M+H]^+$	-1.71	233.1166;275.1637;355.2252	Δ⁹-THCAM A	Cannabinoids
			371.2250	$[M-H]^-$	-5.96	259.1697;303.1596;327.2325		
A9	$C_{20}H_{26}O_4$	65.270	329.1775	$[M-H]^-$	-1.67	151.0767;217.1250;261.1129;283.1715;311.1659	CBDVA	Cannabinoids
			331.1914	$[M+H]^+$	0.25	233.1195;257.1157;313.1801		
A10	$C_{21}H_{30}O_2$	69.692	313.2187	$[M-H]^-$	-4.44	121.0355;162.0610;174.1061;245.1490	CBDA fragment	Cannabinoids
A11*	$C_{22}H_{30}O_4$	69.692	359.2216	$[M+H]^+$	-0.24	219.1003;261.1478;341.2110	CBDA	Cannabinoids
			357.2088	$[M-H]^-$	-4.65	179.1081;245.1550;271.1355;289.1460;313.2183;339.1981		
A12*	$C_{21}H_{30}O_2$	71.044	315.2307	$[M+H]^+$	3.68	135.1153;193.1210;259.1679	CBD	Cannabinoids

			313.2181	$[M-H]^-$	-2.53	107.0519;179.1102;245.1552		
A13	$C_{21}H_{30}O_2$	78.659	313.2200	$[M-H]^-$	-8.58	191.1155;245.1614	CBC	Cannabinoids
A14	$C_{19}H_{26}O_2$	73.009	285.1888	$[M-H]^-$	-9.77	163.0787;217.1300	Δ^9-THCVA fragment	Cannabinoids
A15	$C_{18}H_{22}O_4$	67.974	301.1465	$[M-H]^-$	-6.51	135.0439;189.0949;257.1554	THCA-C1	Cannabinoids
A16	$C_{21}H_{30}O_2$	79.889	313.2191	$[M-H]^-$	-5.75		Δ^9-THC	Cannabinoids
B1	$C_{27}H_{28}O_{17}$	24.127	625.1383<br>623.1272	$[M+H]^+$<br>$[M-H]^-$	-2.61<br>-2.93	287.0539;463.0857<br>285.0403;327.0513;489.1053	Luteolin-*O*-GluA-Glc	Flavonoid-*O*-glycosides
B2	$C_{27}H_{28}O_{16}$	27.812	609.1440<br>607.1333	$[M+H]^+$<br>$[M-H]^-$	-1.66<br>-4.67	271.0590;287.0536;447.0916<br>113.0261;269.0466;285.0515;337.0757;427.0636	Apigenin-*O*-GluA-Glc	Flavonoid-*O*-glycosides
B3*	$C_{21}H_{18}O_{11}$	32.233	447.0920<br>445.0797	$[M+H]^+$<br>$[M-H]^-$	-0.42<br>-4.63	271.0956<br>113.0250;175.0244;269.0458	Apigenin-7-*O*-GluA	Flavonoid-*O*-glycosides
B4*	$C_{21}H_{18}O_{12}$	27.566	463.0866<br>461.0748	$[M+H]^+$<br>$[M-H]^-$	-1.09<br>-4.87	287.0547<br>113.0248;285.0415;357.0601	Luteolin-7-*O*-GluA	Flavonoid-*O*-glycosides
B5	$C_{22}H_{20}O_{12}$	34.690	477.1015<br>475.0900	$[M+H]^+$<br>$[M-H]^-$	-2.63<br>-3.78	301.0702<br>113.0246;175.0249;299.0565	Chrysoeriol-7-*O*-GluA	Flavonoid-*O*-glycosides
B6	$C_{22}H_{20}O_{11}$	42.059	461.1075<br>459.0952	$[M+H]^+$<br>$[M-H]^-$	-0.73<br>-4.16	285.0758<br>85.0301;113.0249;175.0250;283.0614	Acacetin-7-*O*-GluA	Flavonoid-*O*-glycosides
B7	$C_{22}H_{20}O_{12}$	33.584	477.1018<br>475.0905	$[M+H]^+$<br>$[M-H]^-$	-2<br>-4.83	301.0697<br>113.0253;175.0254;299.0570	Chrysoeriol-7-*O*-GluA isomer	Flavonoid-*O*-glycosides
B8	$C_{27}H_{28}O_{15}$	31.742	591.1365<br>593.1493	$[M-H]^-$<br>$[M+H]^+$	-1.61<br>1.35	269.0456;321.0832<br>271.0585;447.0936	Apigenin-*O*-GluA-Rha	Flavonoid-*O*-glycosides
B9	$C_{26}H_{26}O_{15}$	30.759	577.1231	$[M-H]^-$	-5.55	269.0460;307.0667		

			579.1338	[M+H]$^+$	1.12	271.0587;447.0910	Apigenin-*O*-GluA-Xyl	Flavonoid-*O*-glycosides
B10	$C_{26}H_{26}O_{16}$	26.826	593.1182	[M-H]$^-$	-5.71	285.0415;327.0517	Luteolin-*O*-GluA-Xyl	Flavonoid-*O*-glycosides
			595.1290	[M+H]$^+$	0.61	287.0520;463.0850		
B11	$C_{28}H_{30}O_{17}$	30.265	637.1449	[M-H]$^-$	-6.08	299.0574;337.0772	Chrysoeriol-*O*-GluA-Glc isomer	Flavonoid-*O*-glycosides
			639.1564	[M+H]$^+$	-1.29	301.0696;477.1023		
C1	$C_{22}H_{22}O_{11}$	28.917	463.1226	[M+H]$^+$	-1.92	301.0718;313.0699;343.0805;367.0802;397.0909;409.0917;427.1013;445.1121	Chrysoeriol-*C*-Glc	Flavonoid-*C*-monoglycosides
			461.1100	[M-H]$^-$	-2.3	298.0486;341.0679;371.0771		
C2*	$C_{21}H_{20}O_{10}$	25.846	433.1125	[M+H]$^+$	-0.98	283.0593;313.0699;337.0704;367.0794;379.0807;397.0918;415.1025	Vitexin	Flavonoid-*C*-monoglycosides
			431.0986	[M-H]$^-$	-0.53	89.0240;179.0353;311.0562		
C3	$C_{22}H_{22}O_{10}$	34.812	447.1273	[M+H]$^+$	-2.85	285.0744;297.0749;309.0746;327.0851;351.0841;381.0958;393.0949;411.1067;429.1165	Acacetin-*C*-Glc	Flavonoid-*C*-monoglycosides
			445.1158	[M-H]$^-$	-3.99	282.0536;283.0561;285.0414;286.0436297.0763;325.0728;355.0824		
C4	$C_{21}H_{20}O_{11}$	23.267	449.1060	[M+H]$^+$	-4.1	287.0540;299.0541;329.0644;353.0645;367.0802;383.0739;395.0746;413.0850;431.0955	Luteolin-*C*-Glc	Flavonoid-*C*-monoglycosides
			447.0925	[M-H]$^-$	-1.75	297.0427;327.0514;357.0618;393.0540		
D1*	$C_{27}H_{30}O_{15}$	25.601	595.1651	[M+H]$^+$	-1.09	271.0592;313.0702;415.1004;433.1125	Vitexin 4''-*O*-Glc	Flavonoid-*C*-diglycosides
			593.1539	[M-H]$^-$	-4.55	293.0459;413.0893;473.1096		
D2	$C_{27}H_{30}O_{16}$	23.636	611.1595	[M+H]$^+$	-1.9	287.0536;329.0641;413.0864;449.1066	Luteolin-*C*-Glc-*O*-Glc	Flavonoid-*C*-diglycosides
			609.1497	[M-H]$^-$	-5.89	309.0403;327.0518;357.0617;429.0836;489.1040		

D3*	$C_{27}H_{30}O_{14}$	26.338	579.1726	$[M+H]^+$	3.06	271.0600;313.0697;397.0896;415.0990;433.1114	Vitexin-2''-*O*-Rha	Flavonoid-*C*-diglycosides
			577.1573	$[M-H]^-$	-1.77	293.0453;311.0567;413.0893;457.1125		
D4	$C_{28}H_{32}O_{15}$	32.970	609.1793	$[M+H]^+$	-3.45	285.0743;301.0693;327.0847;411.1069;429.1165;447.1272	Acacetin-*C*-Glc-*O*-Glc	Flavonoid-*C*-diglycosides
			607.1654	$[M-H]^-$	2.37	59.0138;71.0134;89.0261;101.0236;119.0355;299.0558;307.0581		
D5	$C_{27}H_{30}O_{15}$	24.004	595.1650	$[M+H]^+$	-1.26	287.0539;329.0636;353.0640;383.0737;413.0839;431.0972;449.1071	Luteolin-*C*-Glc-*O*-Rha	Flavonoid-*C*-diglycosides
			593.1545	$[M-H]^-$	-5.56	309.0396;327.0510;357.0624;429.0841;473.1093		
D6	$C_{28}H_{32}O_{16}$	28.426	625.1762	$[M+H]^+$	-0.18	301.0694;343.0799;397.0887;427.1018;445.1118;463.1231	Chrysoeriol-*C*-Glc-*O*-Glc	Flavonoid-*C*-diglycosides
			623.1640	$[M-H]^-$	-3.59	89.0256;341.0681;443.0999;503.1229		
D7	$C_{28}H_{32}O_{14}$	33.707	593.1854	$[M+H]^+$	-1.83	285.0734;327.0847;351.0855;381.0967;411.1045;429.1165;447.1276	Acacetin-*C*-Glc-*O*-Rha	Flavonoid-*C*-diglycosides
			591.1746	$[M-H]^-$	-4.51	59.0158;89.0233;103.0403;307.0615;325.0714;427.1083		

* identified with the reference substances

Table S10. Investigation of regression equation, linear range, the limit of detection, and limit of quantification of 13 components in *C. sativa* leaves

ID	Calibration Curves	Correlation Coefficient (R^2)	Linear Range (μg/mL)	LOD (ng/mL)	LOQ (ng/mL)
F1	Y=0.5650X-0.0065	0.9960	0.00125-1.000	0.0303	0.1030
F2	Y=0.1661X+0.0173	0.9959	0.00680-5.440	0.6876	1.2500
F3	Y=0.2130X+0.0293	0.9978	0.00656-5.250	0.7219	0.3938
F4	Y=0.3641X+0.0062	0.9982	0.00245-1.960	0.1785	0.2295
F5	Y=0.2239X+0.0834	0.9957	0.0163-13.00	0.0146	0.1733
F6	Y=0.4032X+0.0392	0.9929	0.00260-2.080	0.3860	2.4612
F7	Y=0.3107X+0.0073	0.9931	5.15×10^{-4}-0.4120	0.1391	1.0700
F8	Y=1.3793X-0.1753	0.9976	0.0158-12.625	0.0246	0.1578
F9	Y=3.0614X-0.0427	0.9972	0.00125-0.250	0.0094	0.0156
F10	Y=19.7567X+0.009	0.9985	0.00103-0.2060	0.0028	0.0248
F11	Y=5.9922X+0.1009	0.9976	4.16×10^{-4}-0.6660	0.0021	0.0333
C1	Y=1.7744X-0.0227	0.9920	0.00156-2.500	0.0368	0.0625
C2	Y=2.1709X-0.0209	0.9930	0.00156-2.500	0.0184	0.0313

Table S11. Determination results of 13 chemical constituents in *C. sativa* leaves (μg/g, Mean±SD, n=2)

No.	F1	F2	F3	F4	F5	F6	F7
L01	2.25±0.87	390.38±46.00	152.25±14.52	86.25±8.26	Tr	Tr	Tr
L02	151.8±2.58	6780.42±168.16	3223.78±45.36	600.08±12.51	6.00±0.00	Tr	9.00±0.01
L03	115.62±2.12	9715.50±480.44	1901.65±50.20	706.83±19.59	19.14±0.75	1117.12±28.3	18.02±0.00
L04	7.50±0.00	6061.88±209.05	357.38±10.56	418.88±12.69	10.50±0.00	Tr	4.50±0.00
L05	124.13±5.39	5639.63±202.37	1588.13±51.76	751.88±30.28	11.25±0.87	Tr	6.00±0.00
L06	4.49±0.01	5880.11±284.19	479.43±20.91	610.05±27.18	16.09±0.76	Tr	5.99±0.01
L07	21.75±0.87	13693.43±491.58	883.88±27.26	1251.38±40.46	25.50±1.73	Tr	12.75±0.87
L08	15.38±0.75	3631.13±124.29	815.63±30.97	99.75±4.50	9.00±0.00	Tr	3.38±0.75
L09	7.50±0.00	6703.91±80.15	543.48±2.62	533.74±8.68	10.49±0.01	Tr	7.12±0.75
L10	283.12±7.31	5566.65±265.96	786.34±37.55	552.35±25.51	9.75±0.85	Tr	3.75±0.87
L11	41.23±1.49	5962.40±268.84	811.83±37.08	366.56±17.30	7.87±0.75	Tr	4.50±0.00
L12	159.81±2.25	4532.19±79.65	1306.51±21.08	220.43±3.74	10.48±0.00	Tr	3.74±0.86
L13	126.06±4.49	3047.71±133.34	1036.29±55.00	84.42±4.36	4.50±0.00	Tr	3.00±0.00
L14	97.35±1.67	6185.32±78.94	1082.87±17.57	290.19±3.60	21.34±0.74	Tr	12.73±0.86
L15	127.88±3.94	6106.50±272.90	933.75±34.85	329.63±17.89	20.25±0.87	Tr	8.63±0.75
L16	554.34±25.43	7895.97±422.36	363.20±26.24	951.30±51.21	6.00±0.00	Tr	3.00±0.00
L17	395.43±15.22	6001.17±123.33	357.57±6.84	1028.13±38.40	7.50±0.00	Tr	3.00±0.00
L18	92.72±2.56	3396.77±84.24	649.77±15.81	420.42±15.65	4.50±0.00	Tr	3.00±0.00
L19	248.49±14.77	6078.97±276.16	498.85±27.47	774.75±32.78	9.01±0.01	Tr	3.00±0.00
L20	132.82±3.65	7107.53±86.32	656.20±16.21	620.19±4.79	6.00±0.00	Tr	3.00±0.00
L21	363.75±12.09	7157.25±312.58	250.88±10.70	980.25±37.64	10.50±0.00	Tr	4.13±0.75
L22	22.47±1.22	3727.53±50.72	776.96±19.87	251.99±8.83	1.50±0.00	Tr	1.50±0.00

L23	140.86±4.59	4573.87±80.07	629.00±3.23	415.84±10.17	4.50±0.01	Tr	1.50±0.00
L24	60.00±2.06	3223.75±163.88	308.61±17.80	437.23±23.30	5.62±0.75	33.37±2.21	3.00±0.00
L25	Tr	316.82±17.29	201.20±13.54	17.27±0.87	18.39±0.75	2686.94±106.74	1.50±0.00
L26	176.45±6.40	1959.67±128.59	985.26±70.44	138.24±11.04	125.50±5.66	8265.73±503.13	19.48±1.73
L27	3.00±0.00	601.13±31.62	634.88±30.85	40.13±1.44	46.88±1.44	4056.75±113.14	8.63±0.75
L28	100.83±1.49	2243.60±75.14	958.02±17.67	161.17±4.91	71.96±2.70	4233.44±167.84	10.49±0.01
L29	24.76±1.92	2080.76±75.35	996.48±50.93	114.05±5.13	120.43±4.25	7316.42±272.01	17.26±0.86
L30	5.24±0.87	2918.33±168.41	1429.95±71.21	176.82±7.34	68.93±3.24	6539.46±193.54	10.11±0.75
L31	306.34±44.50	1631.92±99.53	839.94±69.85	113.99±5.99	13.87±0.74	128.99±17.26	3.00±0.00
L32	26.26±0.00	3741.73±223.21	2539.99±140.83	279.14±6.67	46.15±19.30	4001.90±809.06	11.63±3.59
L33	7.50±1.39	1921.38±96.73	1167.98±35.57	62.60±3.54	144.69±1.87	9019.83±186.29	32.24±0.01
L34	43.12±0.88	2910.73±146.23	1030.87±124.85	137.25±10.51	62.25±4.93	6600.64±541.52	7.50±1.43
L35	13.13±2.25	Tr	Tr	Tr	11.25±0.87	372.75±25.05	6.75±0.87
L36	290.71±10.94	Tr	Tr	Tr	61.06±0.75	2259.74±25.28	18.36±0.75
L37	10.87±0.75	Tr	Tr	9.37±1.43	51.35±1.44	1274.01±57.56	17.62±0.76
L38	809.14±48.49	Tr	Tr	Tr	45.68±0.89	1716.06±21.59	42.31±0.76
L39	27.72±2.60	Tr	Tr	Tr	49.45±1.22	1051.20±8.16	36.71±1.50
L40	82.79±2.25	Tr	Tr	Tr	45.33±2.56	1239.64±161.78	46.83±3.09
L41	653.66±45.84	Tr	Tr	Tr	117.36±18.39	3570.79±518.24	77.24±16.57
L42	391.88±12.67	Tr	Tr	Tr	64.88±5.02	2516.92±350.81	46.5±3.73
L43	16.50±0.00	Tr	Tr	Tr	20.63±1.44	991.50±22.68	15.00±0.00
L44	5.25±0.87	Tr	Tr	Tr	16.12±0.75	882.69±27.41	10.49±0.01
L45	837.57±24.46	Tr	Tr	Tr	12.35±0.75	549.78±13.79	10.85±0.75
L46	7.50±0.00	Tr	Tr	Tr	60.72±3.55	2226.42±105.38	44.98±1.23
L47	1317.74±79.05	Tr	Tr	Tr	20.96±2.12	1053.52±92.25	12.35±0.75
L48	16.49±1.22	Tr	Tr	Tr	116.19±5.06	3071.56±125.2	56.22±4.95

L49	Tr	Tr	Tr	Tr	Tr	139.51±6.52	1.50±0.00
L50	40.83±3.94	Tr	Tr	Tr	56.19±0.87	1657.72±20.04	32.22±0.87
L51	115.18±10.78	Tr	Tr	Tr	65.66±4.84	1764.17±94.93	15.38±1.44
L52	11.24±0.85	Tr	Tr	Tr	76.05±2.58	2683.39±73.35	51.70±0.81
L53	28.11±1.45	Tr	Tr	Tr	51.35±0.77	1219.27±7.98	14.99±0.01
L54	934.98±38.74	Tr	Tr	Tr	52.15±1.43	2417.32±32.96	29.26±0.87
L55	Tr	Tr	Tr	Tr	48.70±1.93	2610.76±53.30	7.49±0.00
L56	Tr	Tr	Tr	6.00±1.73	84.00±2.45	2249.25±37.12	12.00±0.00
L57	Tr	Tr	Tr	Tr	73.31±0.78	2059.03±39.27	12.03±0.01
L58	Tr	Tr	Tr	Tr	136.13±2.00	2190.77±35.48	31.12±1.42
L59	Tr	Tr	Tr	Tr	134.22±6.74	5873.66±189.53	20.94±1.20
L60	Tr	Tr	Tr	Tr	111.58±1.96	5154.12±75.42	23.59±0.76
L61	Tr	Tr	Tr	14.62±2.23	66.75±1.90	1925.95±76.12	13.87±0.74
L62	Tr	Tr	Tr	Tr	229.09±9.80	4418.44±174.53	63.09±3.22
L63	Tr	Tr	Tr	Tr	77.44±0.87	5386.83±41.77	28.19±1.42
L64	Tr	Tr	Tr	8.25±0.87	127.94±4.11	9095.27±298.18	26.26±0.85
L65	3.00±0.00	Tr	Tr	18.36±5.65	420.16±12.25	15521.03±843.00	64.84±4.92
L66	3.00±0.00	Tr	Tr	Tr	489.13±4.11	15676.96±114.74	82.46±2.74
L67	4.13±0.87	Tr	Tr	10.88±3.13	231.75±4.15	9517.41±343.31	27.00±0.03
L68	5.99±0.00	Tr	Tr	Tr	206.79±8.02	14934.82±351.17	51.32±0.75
L69	Tr	Tr	Tr	5.63±3.09	42.77±0.84	4332.61±140.44	15.01±0.01
L70	Tr	Tr	Tr	4.50±1.73	105.32±6.11	5441.76±349.14	67.84±1.40
L71	25.49±0.01	Tr	Tr	6.00±0.00	214.77±12.81	7116.78±502.96	29.61±1.45
L72	24.74±0.88	158.17±7.48	206.15±7.90	84.33±3.14	103.45±2.17	7054.46±252.23	39.36±1.46
L73	131.94±4.11	11.99±0.01	15.37±0.76	7.12±0.75	149.55±3.08	6274.70±176.73	39.36±2.56

Table S11 (Continued)

No.	F8	F9	F10	F11	C1	C2
L01	Tr	Tr	28.13±0.75	322.63±25.78	1240.32±74.42	3033.66±135.19
L02	8.25±0.87	Tr	64.09±0.77	475.67±8.64	Tr	381.16±8.55
L03	1132.13±25.69	Tr	61.94±1.44	421.06±20.53	Tr	181.5±7.05
L04	Tr	15.38±0.75	51.75±2.60	959.66±59.24	3849.86±339.44	234.76±14.53
L05	Tr	21.38±0.75	52.50±2.12	1289.09±21.59	6136.37±125.83	381.44±18.73
L06	Tr	4.49±0.01	29.94±1.76	924.03±88.41	2967.13±212.36	210.37±17.97
L07	Tr	35.25±0.87	83.25±5.81	1920.67±65.78	697.17±38.34	78.68±5.43
L08	Tr	19.88±0.75	26.63±0.75	1201.37±16.22	2093.46±187.03	172.87±14.65
L09	Tr	10.49±0.01	44.60±3.12	868.11±69.63	835.22±43.92	84.43±3.90
L10	Tr	8.25±0.86	58.87±2.18	1732.3±87.75	1426.33±125.08	160.88±13.53
L11	Tr	11.99±0.01	19.86±0.74	1215.09±53.18	1109.58±12.01	1774.54±7.58
L12	Tr	4.49±0.00	22.08±0.75	919.20±21.54	1413.32±67.66	114.48±4.23
L13	Tr	15.01±0.01	15.38±1.44	522.11±35.06	1451.57±69.09	1791.33±101.48
L14	Tr	34.82±0.74	31.08±0.74	1287.93±42.70	2115.89±47.71	170.56±1.18
L15	Tr	6.00±0.00	10.5±0.00	660.12±17.11	322.18±10.70	45.44±7.20
L16	Tr	Tr	59.97±0.03	699.17±17.19	4067.70±89.72	3718.88±58.21
L17	Tr	Tr	54.35±0.77	712.92±18.63	7727.20±133.19	417.03±51.43
L18	Tr	Tr	22.15±0.75	459.29±13.61	2780.69±49.63	1339.50±36.98
L19	Tr	Tr	33.03±1.20	431.27±10.57	1336.69±42.33	117.04±3.53
L20	Tr	Tr	33.02±1.71	282.88±4.74	977.42±29.04	74.75±3.46
L21	Tr	Tr	34.13±0.75	437.26±14.32	1201.45±37.44	63.98±2.17
L22	Tr	Tr	25.84±1.45	245.54±19.75	1924.39±69.69	82.37±3.07

L23	Tr	Tr	74.93±3.75	729.60±46.97	1166.91±52.06	950.95±44.67
L24	51.75±1.91	Tr	15.37±0.74	293.84±16.90	1084.89±67.74	746.90±50.80
L25	569.44±28.18	Tr	4.50±0.00	145.22±3.97	103.24±3.35	173.85±8.29
L26	3095.15±59.66	Tr	18.36±1.43	377.55±17.02	404.15±10.29	61.00±0.79
L27	1480.13±62.48	Tr	9.75±0.87	205.75±7.28	551.15±4.05	41.36±0.36
L28	1090.70±11.32	Tr	11.24±0.86	281.55±15.35	271.19±27.98	128.26±18.18
L29	2826.99±123.40	6.00±0.00	6.00±0.00	158.13±5.16	286.04±26.32	174.79±14.49
L30	1412.71±57.05	3.00±0.00	11.99±1.73	229.43±12.98	547.80±13.64	486.32±10.08
L31	31.50±2.70	19.12±0.74	8.25±0.86	183.33±14.63	7.99±0.98	373.23±70.47
L32	1258.37±455.61	7.50±1.43	22.89±2.58	672.79±55.59	814.65±0.94	1233.40±56.12
L33	4492.32±33.81	8.62±1.71	23.24±0.76	320.74±14.05	9.75±0.86	299.74±10.71
L34	1219.49±35.85	16.50±0.00	11.63±3.08	245.04±85.27	23.06±14.21	87.80±71.95
L35	622.50±4.74	Tr	40.13±2.25	350.09±13.34	264.26±8.69	17.66±0.38
L36	1971.28±42.27	3.00±0.00	33.34±3.09	313.64±36.29	15.51±2.00	109.62±18.16
L37	1464.77±45.13	9.00±0.01	32.61±3.96	358.53±33.70	5.79±0.37	118.80±11.29
L38	2979.79±32.73	Tr	26.59±1.45	508.47±22.52	184.7±9.49	143.08±14.09
L39	2163.09±69.26	4.50±0.00	12.74±0.87	211.02±8.42	49.61±4.92	24.88±2.85
L40	3495.25±473.57	32.22±3.57	13.49±0.00	233.88±6.79	83.92±3.96	63.69±1.15
L41	3755.90±332.10	9.00±0.01	22.50±0.03	373.94±17.64	104.04±14.64	11.06±1.56
L42	3018.06±510.39	16.50±1.75	37.13±3.13	499.23±62.77	268.76±37.13	23.52±3.13
L43	1152.00±27.33	9.00±0.00	20.63±0.75	351.68±9.42	6.79±0.55	303.19±23.65
L44	1235.39±28.88	9.75±0.87	10.49±0.01	160.02±4.01	109.95±2.45	50.67±1.12
L45	849.93±22.65	10.48±0.00	16.47±1.73	284.78±19.23	110.22±6.41	13.87±1.45
L46	4289.78±192.03	18.37±0.76	13.12±0.75	253.36±7.13	20.06±0.98	155.77±7.16
L47	1663.92±84.94	4.49±0.00	35.55±3.09	510.77±50.09	94.03±3.71	246.63±9.76
L48	3266.46±126.19	53.97±1.23	21.74±0.87	276.66±11.58	15.17±0.70	101.97±6.01

L49	641.98±24.07	3.00±0.00	29.25±0.90	361.48±10.29	8.8±0.25	459.76±15.68
L50	2546.70±81.50	10.49±0.00	8.99±0.00	198.76±3.92	120.7±2.88	13.88±0.51
L51	975.50±38.46	3.00±0.00	13.51±0.01	191.58±12.47	101.59±2.73	42.88±2.54
L52	3979.98±76.57	15.73±0.87	25.47±0.03	381.10±11.05	282.37±3.73	34.90±0.46
L53	1310.34±9.98	11.99±0.01	35.61±1.45	708.49±16.05	325.04±14.61	340.09±12.61
L54	2212.48±33.04	0.00±0.01	39.02±0.02	427.10±14.79	151.18±45.75	9.71±0.41
L55	1135.49±6.52	14.99±0.00	13.49±0.00	124.73±3.56	59.26±2.05	10.15±0.43
L56	980.63±28.78	20.63±0.75	13.50±0.00	101.51±1.66	1.66±0.04	42.97±0.87
L57	1316.18±31.79	26.69±0.76	15.79±0.86	109.01±2.83	1.61±0.08	130.54±5.25
L58	2217.03±33.85	67.88±0.80	16.50±0.02	165.97±7.64	76.38±1.40	73.98±2.17
L59	2058.99±72.13	7.48±0.01	12.71±0.86	135.09±15.66	59.23±5.13	49.48±3.21
L60	3886.30±41.23	19.10±0.74	11.23±0.86	126.03±6.13	Tr	69.90±2.82
L61	2299.38±137.43	18.75±0.84	31.87±0.73	192.67±9.46	5.28±0.64	126.37±4.46
L62	5754.68±210.28	31.55±1.21	52.20±1.45	352.88±5.51	67.67±1.80	67.66±1.37
L63	3748.11±44.59	24.81±0.87	46.62±1.71	239.06±8.52	Tr	64.33±2.05
L64	3978.45±137.58	6.00±0.00	62.28±4.70	843.28±81.33	64.42±4.56	63.29±4.89
L65	5840.09±560.06	55.85±4.63	117.69±13.2	922.87±144.37	5.55±0.82	115.31±14.78
L66	7700.55±78.04	98.58±1.88	39.36±0.76	493.93±14.27	Tr	76.35±1.41
L67	2634.42±59.58	32.25±0.9	45.00±0.05	366.88±3.49	30.07±0.06	29.95±0.77
L68	6706.54±204.38	88.79±2.56	63.69±6.98	504.52±74.84	5.42±0.71	56.00±8.16
L69	3115.17±27.34	32.64±0.74	29.64±0.74	393.79±19.56	1.97±0.09	222.91±5.71
L70	8275.65±518.64	38.6±2.54	49.47±2.10	493.68±8.69	10.48±0.30	122.34±1.61
L71	5108.45±292.07	41.6±1.90	65.97±3.50	707.82±39.32	8.35±0.51	90.25±5.47
L72	4881.99±128.67	22.11±0.76	22.11±1.45	321.62±10.40	Tr	118.55±2.40
L73	3856.78±146.45	11.24±0.86	17.99±1.23	222.64±18.27	Tr	57.64±5.44

Tr: Trace, the content below the linear range

Table S12. The mean values (n=3) of DPPH (mmol/L), ABTS (mmol/L) and FRAP (mmol/L) of 73 *C. sativa* leaves

No.	DPPH	ABTS	FRAP	No.	DPPH	ABTS	FRAP
L01	0.16	0.16	0.07	L38	0.70	0.77	0.37
L02	0.65	0.58	0.46	L39	0.48	0.45	0.24
L03	0.74	0.62	0.49	L40	0.29	0.26	0.16
L04	0.73	0.74	0.33	L41	0.65	0.70	0.41
L05	0.93	0.75	0.40	L42	0.48	0.42	0.24
L06	0.98	0.91	0.49	L43	0.40	0.34	0.15
L07	1.32	0.98	0.85	L44	0.18	0.27	0.19
L08	0.77	0.61	0.27	L45	0.51	0.36	0.27
L09	0.77	0.92	0.43	L46	0.40	0.34	0.21
L10	0.91	0.94	0.55	L47	0.64	0.61	0.33
L11	0.83	0.69	0.38	L48	0.61	0.47	0.34
L12	0.66	0.78	0.46	L49	0.13	0.23	0.04
L13	0.68	0.65	0.37	L50	1.00	0.75	0.53
L14	0.94	0.71	0.41	L51	0.31	0.30	0.19
L15	0.89	0.80	0.42	L52	0.42	0.39	0.30
L16	0.95	0.66	0.54	L53	0.52	0.57	0.30
L17	0.86	0.67	0.43	L54	0.51	0.56	0.29
L18	0.53	0.52	0.27	L55	0.33	0.15	0.05
L19	0.79	0.55	0.52	L56	0.25	0.10	0.08
L20	0.67	0.48	0.35	L57	0.20	0.15	0.05
L21	1.06	0.72	0.70	L58	0.38	0.26	0.10
L22	0.40	0.40	0.33	L59	0.49	0.29	0.20
L23	0.71	0.53	0.49	L60	0.32	0.18	0.13
L24	0.54	0.40	0.34	L61	0.28	0.19	0.08
L25	0.31	0.19	0.14	L62	0.35	0.25	0.13
L26	0.79	0.69	0.59	L63	0.31	0.22	0.17
L27	0.47	0.27	0.25	L64	0.72	0.50	0.25
L28	0.82	0.59	0.53	L65	0.77	0.43	0.37
L29	0.74	0.45	0.53	L66	0.86	0.47	0.56
L30	0.92	0.55	0.60	L67	0.81	0.49	0.40
L31	0.82	0.53	0.47	L68	0.90	0.50	0.56
L32	0.62	0.48	0.33	L69	0.26	0.24	0.23
L33	0.90	0.57	0.63	L70	0.45	0.42	0.24
L34	0.95	0.70	0.69	L71	0.46	0.36	0.27
L35	0.05	0.21	0.09	L72	0.86	0.58	0.59
L36	0.44	0.34	0.26	L73	0.71	0.49	0.56
L37	0.26	0.30	0.15				

Table S13. The correlation between content and total antioxidant activity in *C. sativa* leaves

correlation	FRAP	ABTS	DPPH
Total flavonoids	0.579**	0.322**	0.563**
Total cannabinoids	0.189	0.365**	0.314**

** P$<$0.01, significantly correlated

Table S14. Correlation coefficient between quantitative components and activity in *C. sativa* leaves

Analyte	FRAP coefficient	ABTS coefficient	DPPH coefficient	VIP
(-)-Epicatechin	0.001	0.413	0.224	0.383
Apigenin -7-*O*-glucuronide	0.006	0.270	0.158	0.860
Diosmetin 7-*O*-glucoside	0.001	0.002	0.000	0.005
Cannflavin B	0.002	0.005	0.001	0.010
Cannflavin A	0.039	0.495	0.306	0.519
Vitexin-4''-*O*-glucoside	0.437	0.44	0.659	2.849
Vitexin-2''-*O*-rhamnoside	0.072	0.113	0.132	0.441
Vitexin	0.041	0.021	0.051	0.262
Luteolin-7-*O*-glucuronide	0.244	0.006	0.281	1.685
Luteolin 7-*O*-glucoside	0.004	0.004	0.003	0.032
Apigenin -7-*O*-glucoside	0.000	0.015	0.008	0.016
CBD	0.082	0.394	0.289	0.747
Δ^9-THC	0.011	0.239	0.121	0.255

Disclaimer/Publisher's Note: The statements, opinions and data contained in all publications are solely those of the individual author(s) and contributor(s) and not of MDPI and/or the editor(s). MDPI and/or the editor(s) disclaim responsibility for any injury to people or property resulting from any ideas, methods, instructions or products referred to in the content.
